# Experimental design for the optimization of propidium monoazide treatment to quantify viable and non-viable bacteria in piggery effluents

**DOI:** 10.1186/s12866-015-0505-6

**Published:** 2015-08-16

**Authors:** Jérémy Desneux, Marianne Chemaly, Anne-Marie Pourcher

**Affiliations:** IRSTEA, 17 avenue de Cucillé, 35044 Rennes, France; Université Européenne de Bretagne, Rennes, France; French Agency for Food Environmental and Occupational Health Safety, Anses, Laboratory of Ploufragan-Plouzané, F-22440 Ploufragan, France

## Abstract

**Background:**

Distinguishing between viable and dead bacteria in animal and urban effluents is a major challenge. Among existing methods, propidium monoazide (PMA)-qPCR is a promising way to quantify viable cells. However, its efficiency depends on the composition of the effluent, particularly on total suspended solids (TSS)) and on methodological parameters. The aim of this study was evaluate the influence of three methodological factors (concentration of PMA, incubation time and photoactivation time) on the efficiency of PMA-qPCR to quantify viable and dead cells of *Listeria monocytogenes* used as a microorganism model, in two piggery effluents (manure and lagoon effluent containing 20 and 0.4 TSS g.kg^−1^, respectively). An experimental design strategy (Doehlert design and desirability function) was used to identify the experimental conditions to achieve optimal PMA-qPCR results.

**Results:**

The quantification of viable cells of *L. monocytogenes* was mainly influenced by the concentration of PMA in the manure and by the duration of photoactivation in the lagoon effluent. Optimal values differed with the matrix: 55 μM PMA, 5 min incubation and 56 min photoactivation for manure and 20 μM PMA, 20 min incubation and 30 min photoactivation for lagoon effluent. Applied to five manure and four lagoon samples, these conditions resulted in satisfactory quantification of viable and dead cells.

**Conclusion:**

PMA-qPCR can be used on undiluted turbid effluent with high levels of TSS, provided preliminary tests are performed to identify the optimal conditions.

## Background

Accurate and reliable detection of viable pathogenic bacteria in complex matrices like manure and biosolids is a major challenge. Few molecular methods have been developed to differentiate viable from dead cells. Reverse transcription-PCR (RT-PCR) has been used to study the behavior of viable pathogenic bacteria in manured soil [1, 2] and in sludge [3]. However, its use is limited by the difficulty in extracting high quality RNA, by the instability of RNA, and by variations caused by the physiological condition of the cells [4]. The Live/Dead BacLight viability assay can detect viable bacteria but this microscopic method is not suitable for environmental matrices due to interactions between the dye and organic matter [5]. Ethidium monoazide (EMA) or propidium monoazide (PMA) coupled with real-time quantitative PCR (qPCR) have been used to distinguish viable from dead bacteria [6, 7]. These DNA intercalating dyes selectively enter cells with compromised membrane and bind covalently to DNA after photoactivation, thus preventing subsequent PCR amplification [8, 9]. Comparative studies showed that PMA outperformed EMA in the selective removal of dead cells [8, 10, 11]. Despite its advantages over EMA, there is evidence that PMA has limitations when applied to complex environmental matrices [9, 12, 13]. High levels of total suspended solids (TSS) or biomass in sludge or in water samples appeared to interfere with the ability of the PMA-qPCR method to quantify viable bacteria [14]. Indeed, the high organic matter content in sludge and manures and the high turbidity of these matrices may interfere with the photoactivation process. In addition, organic matter provides chemically active cation exchange sites that may retain the PMA and consequently reduce the available concentration of dye [15]. Questions therefore still remain about the applicability of PMA in such matrices.

Several methodological parameters may affect PMA efficiency. Among them, the concentration of the dye, the length of light exposure and of incubation appear to be key parameters [12] that require optimization for the reliable quantification of dead microorganisms. Studies on the use of PMA to quantify non-viable bacteria in effluents are rare and are all based on the works of Nocker et al. [9, 16, 17] and Luo et al. [18]. In these studies, the samples were exposed to a 600-650-W halogen light source placed at a distance of 20 cm from the tubes and light exposure time ranged from 1 to 20 min depending on the experiment [19–21]. The incubation time in the dark also varied, ranging from five [9, 14, 19, 20] to 10 min [22], while PMA concentrations ranged from 2 μM [19] to 300 μM [14]. Due to the lack of data on livestock and urban effluents, the optimal conditions of the PMA application are still not clear and require further investigation.

As a model for the present study, we used *Listeria monocytogenes,* a pathogen that has been reported to enter a viable but nonculturable (VBNC) state [23, 24] and whose presence has been reported in both urban and animal effluents [5, 25]. We determined the optimal conditions of the PMA-qPCR method in two types of piggery effluents that differed in their chemical and physical properties: raw manure and biologically treated liquid manure (lagoon effluent). To this end, we compared the quantification of living (viable) and heat-killed (dead) cultured *L. monocytogenes* cells using PMA-qPCR, qPCR and a cultural method. To simultaneously study the effect of (i) the concentration of PMA, the length of light exposure and of incubation in the dark and (ii) the interaction of these three factors, a response surface method based on a Doehlert design and a desirability function were applied to identify the optimal conditions for the quantification of VBNC cells of *L. monocytogenes*. To check the applicability of the method, the optimal conditions we identified were tested on nine piggery effluents.

## Results

### Use of the desirability approach to determine the optimal conditions for PMA pretreatment

We studied the concentration of PMA, incubation time and photoactivation time in a raw manure and in a lagoon effluent using an experimental design matrix based on a Doehlert design (Table 1). The lowest Δ_dead_ values were 1.2 in the lagoon effluent and 2.4 log_10_ cfu-eq in the manure. It is noteworthy that the maximum theoretical Δ_dead_ value (4.1 in manure and 3.4 in lagoon) depended on the concentration of inoculated cells and on the limit of quantification of the qPCR. This maximum value was reached for 5 and 10 combinations of factors in manure and lagoon effluent, respectively. The values of Δ_viable_ ranged from 0.2 to 1.3 log_10_ cfu-eq in manure and from 0.1 to 1.0 log_10_ cfu-eq in lagoon effluent, demonstrating that the combination of the three methodological factors affected the efficiency of the PMA-qPCR.

**Table 1:** Design matrix of the Doehlert uniform shell design for 3 factors and corresponding responses

Run	Factors			Responses (log_10_ cfu-eq)			
	PMA concentration (μM)	Incubation time (min.)	Photoactivation time (min.)	Manure		Lagoon	
				Δ_viable_	Δ_dead_^b^	Δ_viable_	Δ_dead_
1^a^	160	17.5	29	1.2	3.4	1.0	3.4
2	160	17.5	29	0.9	3.4	1.0	3.4
3	160	17.5	29	1.1	3.4	1.0	3.4
4	160	17.5	29	1.1	3.4	1.0	3.4
5	300	17.5	29	1.6	4.1	1.2	3.4
6	20	17.5	29	0.2	2.7	0.1	3.4
7	230	17.5	56	1.3	4.1	1.2	3.4
8	90	17.5	2	0.6	2.4	0.4	2.5
9	230	17.5	2	1.2	2.4	0.3	2.5
10	90	17.5	56	0.6	3.1	1.0	3.4
11	230	30	38	1.0	4.1	0.6	3.4
12	90	5	20	0.6	2.9	0.3	1.2
13	230	5	20	1.2	3.3	0.7	3.4
14	160	5	47	0.8	4.1	0.8	3.4
15	90	30	20	0.5	3.2	0.7	3.4
16	160	30	11	1.0	4.1	0.6	3.4

^a^The central run (1 to 4) has been repeated 4 times in order to calculate residual variance

^b^The concentrations of the inoculated cells were 8.3 log_10_ cfu mL^−1^ in the manure and 7.3 log_10_ cfu mL^−1^ in the lagoon resulting in a maximum theoretical Δ_dead_ value of 4.1 and 3.4, respectively

The minimum value of Δ_viable_ was observed for a PMA concentration of 20 μM, an incubation time of 17.5 min and a photoactivation time of 29 min in both matrices. Under these conditions, the Δ_dead_ value reached the limit of quantification in the lagoon but was 2.7 log_10_ cfu-eq in the manure, indicating an underestimation of 1.4 log_10_ cfu-eq of the dead cells in this matrix.

**Fig. 1:**
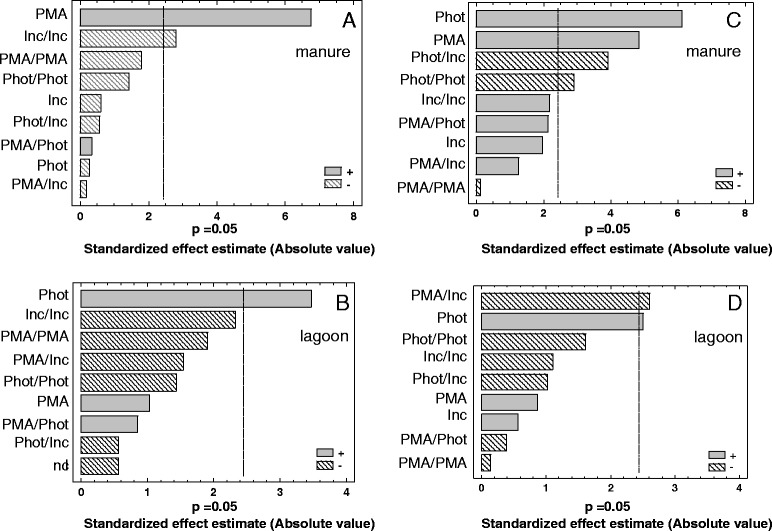
Pareto chart of the individual, quadratic and interactive effect of PMA concentration (PMA), incubation time (Inc) and photoactivation time (Phot) on the Δ_viable_ (**a** and **b**) and Δ_dead_ (**c** and **d**) values in samples of manure and lagoon effluents

The estimated effects of the three independent factors with their second-order interactions on the Δ_viable_ and Δ_dead_ responses are presented on the standardized Pareto charts in Fig. 1, which graphically displays the magnitude of the effects according to the statistical analysis of the experimental data. The effects are ranked in descending order. Analysis of the data showed that the significance of the variables depends on the nature of the matrix. When viable *L. monocytogenes* was inoculated in manure, the PMA concentration were the most important factor affecting the Δ_viable_ values (Fig. 1a). The two other factors had little or no significant effect on the Δ_viable_ values. On the other hand, when viable bacteria were inoculated in the lagoon, only the length of the photoactivation period had a positive effect on the Δ_viable_ values (Fig. 1b). The interpretation of the significance of the factors obtained with the dead cells was more difficult given the high number of similar responses corresponding to runs in which the limit of quantification of the qPCR was reached. This may explain the low significance of the three factors observed in the lagoon effluent (Fig. 1d) and the high number of factors and interactions influencing the Δ_dead_ values in manure (Fig. 1c). Nevertheless, overall, the reduction in Δ_viable_ and the increase in Δ_dead_ values were mainly affected by the concentration of PMA and /or the length of the photoactivation period, whereas incubation time had less impact.

According to the results of the Doehlert design, it is clear that (i) the type of matrix played a role in the effect of the factors and (ii) the Δ_viable_ and Δ_dead_ responses were contradictory. In order to find the best compromise between the two responses, a composite desirability function was calculated by simultaneous minimization of the Δ_viable_ response and maximization of the Δ_dead_ response. Statgraphics Centurion XVI software was used to calculate the optimal conditions. The results of the predicted values of the desirability function for each run of the Doehlert design are listed in Table 2. The three factors needed to maximize desirability for the manure (run 14) differed from those needed for the lagoon effluent (run 6). The optimal conditions to achieve the highest value of the overall desirability function were 55 μM PMA, 5 min incubation and 56 min photoactivation for manure and 20 μM PMA, 20 min incubation and 30 min photoactivation for lagoon effluent, all of which were computed by Statgraphics Centurion XVI software.

**Table 2:** Predicted values of the desirability function at each point conditions set for manure and lagoon

Run	Predicted value	
	Lagoon	Manure
1 to 4	0.426	0.464
5	0.454	0.173
6	0.819^a^	0.411
7	0.000	0.498
8	0.609	0.000
9	0.659	0.174
10	0.605	0.508
11	0.602	0.600
12	0.456	0.475
13	0.596	0.364
14	0.494	0.705^a^
15	0.721	0.678
16	0.754	0.652

^a^The numbers in bold indicate the run corresponding to the higher overall desirability

### Applying the optimal conditions to manures and lagoon effluents

The optimal conditions determined by the desirability function were applied to five manures and four lagoon effluents inoculated with viable and heat-killed *L. monocytogenes* cells to estimate the effect of the matrices on the Δ_viable_ and the Δ_dead_ responses with the same PMA pretreatment. The physico-chemical characteristics and responses are listed in Table 3. The Δ_viable_ responses ranged from 0.12 to 0.83 log_10_. Regardless of the matrix, seven of the nine Δ_viable_ responses resulted in slight underestimation of the viable *L. monocytogene*s cells (less than 0.5 log_10_ cfu-eq). In seven of the nine samples, the observed Δ_dead_ were close to the theoretical maximum value of Δ_dead_ (3.2 and 3.5 log_10_ cfu-eq for manures and lagoons, respectively)_,_ suggesting the underestimation of heat-killed cells was very small. Two manure samples (M3 and M4) had a lower Δ_dead_ value than those of the other samples, resulting in a 0.7 and 0.8 log_10_ underestimation of dead cells, respectively. As, except for samples M3 and M4, the Δ_dead_ responses were close to the limit of quantification, only the relationships among Δ_viable_ responses and physico-chemical parameters were analyzed. The turbidity of the lagoons was positively correlated with Δ_viable_ (Spearman’s ρ = 1.0; *p* < 0.0001) whereas none of the factors were correlated with Δ_viable_ in manure, confirming that turbidity played a major role in the less turbid matrix.

**Table 3:** Physico-chemical parameters and Δ_viable_ and Δ_dead_ responses (expressed in log_10_ cfu-eq) after PMA pretreatment of 5 manures and 4 lagoon effluents

Matrix	Sample	Δ_viable_ mean ± SD^b^	Δ_dead_^a^ mean ± SD	pH	Turbidity (NTU)	VS (g.kg^−1^)	TSS (g.kg^−1^)
manure	M1	0.15 ± 0.2^C^	2.97 ± 0.3^A^	7.6	2612	6.3	3.8
	M2	0.83 ± 0.1^A^	3.25 ± 0.0^A^	8.2	2660	7.6	5.9
	M3	0.49 ± 0.2^ABC^	2.51 ± 0.3^B^	7.3	4624	22.8	14.8
	M4	0.29 ± 0.3^BC^	2.40 ± 0.2^B^	7.5	3323	19.8	12.9
	M5	0.21 ± 0.1^C^	2.93 ± 0.2^A^	7.6	3580	12.4	10.5
	Mean	0.39 ± 0.3	2.81 ± 0.4	7.7 ± 0.3	3360 ± 821	13.8 ± 7.3	9.6 ± 4.7
Lagoon	L6	0.12 ± 0.1^C^	3.35 ± 0.0^A^	8.5	102	1.6	0.9
	L7	0.36 ± 0.1^BC^	3.35 ± 0.0^A^	8.4	120	1.2	0.55
	L8	0.46 ± 0.1^BC^	3.35 ± 0.0^A^	8.4	461	1.8	1.1
	L9	0.69 ± 0.1^ABC^	3.12 ± 0.3^A^	8.4	772	1.3	1.0
	Mean	0.41 ± 0.2	3.29 ± 0.2	8.4 ± 0.0	364 ± 318	1.5 ± 0.3	0.9 ± 0.2

The values followed by different capital letters are statistically different according to a Student-Newman-Keuls test

^a^The concentrations of the inoculated cells were 7.4 log_10_ cfu mL^−1^ in the manure and 7.5 log_10_ cfu mL^−1^ in the lagoon, resulting in a maximum theoretical Δ_dead_ value of 3.2 and 3.5, respectively

^b^mean of triplicates ± standard deviation

## Discussion

The DNA-intercalating agent PMA was used in combination with qPCR for the detection of the viability of bacterial cells. This rapid method does not require cell culture and is consequently particularly attractive for the detection of pathogenic bacteria that can enter a viable but nonculturable (VBNC) state such as *L. monocytogenes* [23, 26]. PMA has been used on a wide range of microorganisms (vegetative and spore forming bacteria, fungal cells, viruses, protozoa) and successfully tested on simple matrices such as broth culture or water samples [19, 27–34]. However, the complex nature of environmental matrices (e.g. urban effluent, sludge, manure) has been shown to influence the effectiveness of the PMA-qPCR [14, 15, 19, 20, 35, 36]. Factors such as turbidity and the concentration of solids [4, 15, 16, 18–21], pH [37], and of organic matter [35, 38] may reduce the efficiency of dye pretreatment. Indeed, the presence of solids and organic matter in urban effluents and sludge interferes with the penetration of PMA into microorganisms with compromised cell membrane integrity [14, 19–22], resulting in underestimation of the number of dead cells. One suggested way of overcoming this bias is diluting the environmental matrices. However, this reduces the concentration of the target organism and, as a result, reduces the sensitivity of the method.

Although the efficiency of PMA-qPCR, PMA may also be affected by methodological factors such as the concentration of PMA, the length of photoactivation and incubation [9, 14, 19, 20], the combination of the three factors tested on effluents and sludge in published literature were mostly similar (i.e. PMA 100 μM, incubation time 5 min and photoactivation time 2 to 5 min).

The results of our experiments performed on raw pig manure, a brown matrix containing 20 g L-1 TSS and lagoon effluent, which was less colored and contained about 50 times less TSS, revealed that the efficiency of the PMA treatment is influenced by the type of effluent and thus require optimization of the methodological factors. The optimal combination of the three factors obtained with the desirability function clearly differed between the manure and the lagoon samples. The analysis of the data of the Doehlert design identified the concentration of PMA in the manure and the photoactivation time in the lagoon effluent as the factors that most influenced Δ_viable_ responses, whereas incubation time was less significant. These results suggest that in turbid matrices such as manure or sludge, the photoactivation time plays a minor role compared to the concentration of PMA and vice versa in less turbid matrices.

The significance and the interaction of factors affecting the Δ_dead_ responses were less clear due to the high limit of quantification of the qPCR, which was reached in 76 and 38 % of the experimental runs in lagoon effluent and in manure, respectively (Table 1). Experiments performed in broth or water with a pure culture of dead cells resulted in a reduction of the cell quantification of between 3 and 5 log_10_ after PMA or EMA pretreatment [14, 17, 31, 32, 39, 40]. In complex matrices, the rate of reduction was lower, around 3 log_10_ [14, 19, 20] or was not satisfactory [20] due to the presence of PCR inhibitors and of solids, which react with the PMA. In our study, when the optimal conditions provided by the desirability function were applied in nine piggery effluent samples, the Δ_viable_ and Δ_dead_ values were generally close to their target values.

As the quantification of dead cells depends on the activation of the acid nucleic-bound dye, quantification could be improved by replacing the halogen lamp used in this study by 460 nm LEDs, which have the advantage of emitting light at a wavelength close to the maximum absorption of PMA (456 nm) and do not generate heat [27]. Moreover, it should be noted that the length of the target gene may also affect the effectiveness of the dye. Longer amplicons appear to be more appropriate than shorter ones to reflect the extent of cell death induced by the PMA or EMA-pretreatment [31, 40–47]. However, amplification of long fragments (longer than 400 bp) increases the likelihood of the formation of secondary structures, thereby reducing PCR efficiency. In our study, the small size of the target fragment (113 bp), does not appear to have negatively influenced the quantification of the dead cells, as most of the nine signals were very close to threshold of the qPCR.

The efficiency of the PMA pretreatment also depends on the target microorganism, which may complicate the selection of the optimal combination of factors [43, 45]. Furthermore, factors such as the ratio of dead cells to viable cells may influence the efficiency of the PMA pretreatment [11, 14, 31]. It should be noted that the piggery effluents tested in our study probably contained a substantial number of cells with damaged membranes that may incorporate PMA and hence interfere with the quantification of the target amplicon after PMA treatment.

In our study, the efficiency of the PMA pretreatment was not impacted by the range of pH (7.3 to 8.5), but depended on the type of effluents, which mainly differed in their TSS contents and turbidity (Table 3). Bae and Wuertz [19] and Taskin et al. [22] reported that TSS contents > 1 g L^−1^ and 4 g L^−1^, respectively, interfered with the cross-linking of PMA, leading to underestimation of heat-treated cells. Wagner et al. [20] reported that the black color of digested sludge limited light penetration thus preventing the crosslinking step of the dye. Similarly, Li et al. [21] pointed out that sludge inhibits the efficiency of PMA due to the presence of dark particles or inhibitory substances. Data on PMA reported by Luo et al. [18] suggest that turbidity has an effect on PMA treatment when the turbidity of samples is more than 10 NTU. However, our data showed that even when turbidity ranged from 100 to 4600 NTU, PMA adequately distinguished between viable and dead cells of *L. monocytogenes*.

In our study, as the optimal combination of the three factors was estimated taking into account the difference in composition of the two matrices, the variations in the TSS levels in the samples of manure (ranging from 6 to 23 g L^−1^) and in samples of lagoon effluent (1.3 to 1.8 g L^−1^ ) had no significant impact on the Δ_viable_ values. Nevertheless, the two manures with the highest level of TSS led to the greater underestimation of the number of dead cells.

## Conclusion

In our experimental conditions (small target amplicon, high level of TSS and high turbidity, use of a halogen lamp to activate the dye), the use of the two combinations of factors selected according the type of matrix based on the desirability function, allowed us to accurately estimate the number of viable and dead cells of *L. monocytogenes* in both manure and lagoon effluent. Our results confirm that PMA-qPCR can be used on undiluted turbid effluent provided that preliminary tests are performed to estimate the optimal conditions for a given matrix.

## Methods

### Bacterial strain and culture conditions

A strain of *L. monocytogenes*, originally isolated from pig manure, was used throughout this study. Stock culture was stored at −80 °C. Cells were cultivated for 24 h at 37 °C in nutritive broth (OXOID) supplemented with 0.3 % glucose (NBG medium) to obtain stationary-phase cultures. Prior to exposure assays, exponential-phase cultures were prepared by inoculating 100 mL of NBG with 150 μL of a stationary-phase culture NBG and incubating the resulting culture at 37 °C for 17 h. To ensure that the exponential phase was reached, viable cells were quantified both by culture and qPCR targeting *hlyA* gene of *L. monocytogenes*.

### Samples of livestock effluent

The experimental design was applied on two types of livestock effluent: raw manure (henceforth referred to as ‘manure’ samples) and lagoon effluent collected from a piggery located in Brittany (France). Raw manure was stored in a tank. After centrifugation, the liquid phase was treated by aerobic digestion. The biologically treated manure was then stored in a settling tank. The liquid supernatant was sent to a lagoon. Manure and lagoon samples (10 l) were collected and transferred to a one liter flask. The flasks were then transferred to the laboratory and stored at 4 °C until physical-chemical characterization and inoculation. The pH of the manure was 7.7 and the lagoon pH was 8.9. Total suspended solids (TSS) in the manure were 20.5 g.kg^−1^ and 0.4 g.kg^−1^ in the lagoon.

Before inoculation, 150 mL of manure and lagoon were placed in a flask and stirred with a magnetic stirrer for 15 min.

### Preparation of viable and dead cells

To maximize the density of viable cells (i.e. 100 % of viable cells), exponential phase cultures of *L. monocytoge*nes grown in NBG were used to inoculate the manure and the lagoon effluent. To generate a population of dead cells (cells with compromised membrane integrity), 5 mL of the exponential phase cultures were transferred in a 12 mL tube immersed in hot water bath at 90 °C for 15 min. The cell suspensions were cooled to ambient temperature before use. The viability of the cells was estimated by plating the *L. monocyto*genes suspensions on TSYE (OXOID) agar, and incubating them at 37 °C for 24 h. Results are expressed as colony forming units (cfu) per mL. The absence of viable cells of *L. monocytogenes* was confirmed by spreading 0.1 mL aliquots of heat-killed cells on TSYE agar (OXOID) which was then incubated for 48 h at 37 °C. Aliquots of heat-killed and viable cells containing 10^7^–10^8^ cells per mL were inoculated (1/10 v/v) in the manure and in lagoon samples described above.

### DNA extraction and quantification

To study the effect of PMA, DNA from viable and heat-killed *L. monocytogenes* inoculated in manure and in lagoon samples, was extracted using a Nucleospin kit for soil (Macherey Nagel) according to the manufacturer’s instructions except that a FastPrep-24 instrument was used to lyse cells (MP Biomedicals) and a minor modification was made in the elution step. The eluent was split into four successive elutions (each performed with 25 μL of elution buffer). The Nucleospin kit was chosen for its ability to extract high quality DNA in large quantities from manures [48]. The primers and the probe targeting the *hlyA* gene (listed in Table 4), along with the quantification procedure for *L. monocytogenes* are described in Nogva et al. [49]. PCR was performed using the ep*Motion*®5070 pipetting system (Eppendorf). Amplification reactions (25 μL) contained 200 μM of dNTP; 0.1 μM specific probe of *L. monocytogenes*; 0.3 μM of each specific primer of *L. monocytogenes*, 12.5 μl of IQ Supermix (Biorad) and 2 μl of 1/10 diluted DNA. The cycling parameters were as follows: 95 °C for 10 min, followed by 40 cycles at 95 °C for 20 s and at 60 °C for 60 s. PCR amplification of 113 bp was carried out in a CFX96 real-time PCR machine (Bio-Rad Laboratories). Real time PCR results were analyzed using the CFX manager software Version 1.1 (Bio-Rad Laboratories). The PCR standard curve was prepared by 10-fold dilution of bacterial genomic DNA extracted from the pure culture of *L. monocytogenes* with the Wizard genomic DNA purification Kit (Promega) according to the manufacturer’s instructions. As *hlyA* is present as a single copy in the genome of *L. monocytogenes* [50], the results are expressed as cfu equivalent (cfu-eq). Dilutions ranged from 4.5 × 10^8^ to 4.5 × 10^0^ cfu-eq. Standard curves were generated by plotting threshold cycles (Ct) against cfu-eq. The quantification limit was 4.2 log_10_ cfu-eq mL^−1^ for the manure and 3.9 log_10_cfu-eq mL^−1^ for the lagoon effluent.

**Table 4:** Primers and probe used for *L. monocytogenes* quantification

Probe or primer	Sequence (5′–3′)	Denaturation temperature (°C)
Primers		
Forward	TGC AAG TCC TAA GAC GCC A	60.3
Reverse	CAC TGC ATC TCC GTG GTA TAC TAA	60.3
Probe	CGA TTT CAT CCG CGT GTT TCT TTT CG	70.2

### Propidium mono azide treatment

Propidium mono azide (PMA) stock solution (20 mM, Biotium) was stored at −20 °C in the dark. The stock solution was transferred into highly light transparent 12 mL polypropylene tubes (Greiner Bio-one) containing either 400 μL of manure or 600 μL of lagoon samples spiked with suspensions of viable or heat-killed cells. After incubation in the dark, the tubes were placed horizontally on ice at a distance of about 20 cm from a 650 W halogen lamp (Inspecktor, ref 100512, R75 118 mm) as recommended by the manufacturer. After photo-induced crosslinking, 250 μL and 500 μL of PMA-treated manure and lagoon samples, respectively, were transferred to a 2 mL Eppendorf tube and centrifuged for 5 min at 5000 *g*. The supernatant was removed and the pellet was immediately placed at −20 °C. The concentrations of the PMA, the incubation time in the dark and under light are mentioned below.

### Experimental shell designs

Statistical analyses were performed using Statgraphics Centurion XVI software (StatPoint Technologies, Inc.). Experiments were conducted following a Doehlert experimental uniform shell design, displaying a uniform distribution of the points on a spherical shell and allowing a number of distinct levels for each factor [51]. Three factors were studied. The minimum and maximum values of each factor were selected according to values reported in the literature. The concentration of PMA in the two matrices was studied at five levels (20, 90, 160, 230, 300 μM), three different incubation times (5, 15, 30 min) and seven photoactivation times (2, 11, 20, 29, 38, 47, 56 min) were studied. The number of experiments required (N) is given by N = k^2^ + k + 1 + n, where k is the number of factors (k = 3) and n is the number of replications at the center of the experimental domain (*n* = 3). Replicates at the central level of the factors were performed to validate the model by means of an estimate of experimental variance. Sixteen experiments were carried out per matrix (manure and lagoon) and per physiological state (viable or heat-killed) leading to a total of four experimental designs.

To optimize PMA pretreatment, it is important to note that under certain conditions, PMA (i) may enter viable cells (leading to underestimation of viable bacteria) and (ii) may react with the organic matrix and consequently not penetrate all the dead cells (leading to underestimation of the dead bacteria). Thus, the optimal conditions determined by the experimental design must maximize penetration of dead cells by PMA and minimize entry into viable cells.

The efficiency of the PMA pretreatment on the quantification of viable and dead cells was estimated by two calculated values: Δ_viable_, Eq. (1) and Δ_dead,_ Eq. (2).

1 $$ {\Delta}_{\mathrm{viable}}={ \log}_{10}\mathrm{C}-{ \log}_{10}\mathrm{X} $$

2 $$ {\Delta}_{\mathrm{dead}} = { \log}_{10}{\mathrm{C}}_{\mathrm{heat}}-{ \log}_{10}\mathrm{X} $$

where C is the concentration of the inoculated cultivable cells, expressed in cfu. mL^−1^, C_heat_, the concentration of the inoculated cultivable cells before heat treatment, expressed in cfu. mL^−1^ and X, the concentration of cells estimated by qPCR after PMA pretreatment (expressed in cfu-eq. mL^−1^).

As the first term of both equations was a constant, we assumed that (i) the higher the Δ_viable_ value, the greater the underestimation of the number of viable cells by the PMA-qPCR and (ii), the lower the Δ_dead_ value, the greater the underestimation of the number of dead cells by the PMA-qPCR.

In the experiments carried out on *L. monocytogenes* inoculated as exponential phase cells, we considered that the difference between the number of cultivable cells per mL and the number of cells per mL measured by qPCR after PMA treatment (Δ_viable_) had to tend to zero, whereas in the experiments performed with dead cells, the difference between the number of cultivable cells (before heat treatment) and the number of cells measured by qPCR after PMA treatment (Δ_dead_) had to be as high as possible. According to the concentration of viable cells inoculated in the matrices (8.3 log_10_ cfu mL^−1^ in manure and 7.3 log_10_ cfu mL^−1^ in lagoon samples) and the limit of quantification of the qPCR for each matrix, Δ_dead_ had to tend to 4.1 log_10_ cfu-eq. mL^−1^ in manure and to 3.4 log_10_ cfu-eq. mL^−1^ in lagoon samples.

To identify the factor settings that optimize the two responses simultaneously (i.e. minimize Δ_viable_ and maximize Δ_dead_) the data was analyzed by combining the individual responses into a desirability function, as proposed by Derringer and Suich [52]. The responses were transformed into a dimensionless desirability scale (d_i_) defined as a partial desirability function ranging from 0 (for a completely undesirable response) to 1 (when the response reached the target value). Once the function was defined for each response, an overall objective function (D), representing the global desirability function, was calculated by determining the geometric mean of the individual desirability. D can be calculated by the expression D = (d_1_^p1^ · d_2_^p2^ · …d_m_^pm^)^1/m^ where *m* is the number of responses and pm their associated weight. The weight of the response represents the relative importance of each individual function d_i_ and may range between 0.1 and 10. With a weight of 1, d_i_ varies in a linear way. In our study, weights equal to 1 were selected. The criteria for optimization of individual responses are listed in Table 5.

**Table 5:** Criteria for multivariate optimization of individual responses

Matrix	Response	Target	Lower limit	Upper limit	Weight	Importance
Manure	Δ_viable_^a^	Target = 0	0.2	1.6	1	3
	Δ_dead_^b^	Target =4.1	2.4	4.1	1	3
Lagoon	Δ_viable_	Target = 0	0.1	1.2	1	3
	Δ_dead_	Target =3.4	1.2	3.4	1	3

^a^difference between the number of cultivable cells and the number of cells measured by qPCR after PMA treatment

^b^difference between the number of cultivable cells (before heat treatment) and the number of cells measured by qPCR after PMA treatment

### Application of the PMA pretreatment on piggery effluents

The optimal conditions determined by the experimental design were tested on nine livestock effluents collected from piggeries located in Britany: five raw manures and four lagoon effluents. Turbidity, pH, volatile solids (VS) and total suspended solids (TSS) were performed on the nine effluents. Each sample was inoculated in triplicate with exponential phase cells of heat-killed or living *L. monocytogenes* cells at a concentration of 2.5 × 10^7^ cfu mL^−1^ in the manures and of 3 × 10^7^ cfu mL^−1^ in the lagoon effluents. To compare the Δ_viable_ and Δ_dead_ values among the effluents tested, the PMA pretreatment with the optimized conditions was applied, as described above.

Correlations of the physical-chemical parameters with the efficiency of PMA were analyzed using a repeated-measures one-way ANOVA followed by a Student–Newman–Keuls test in an all pairwise fashion. Bacterial concentrations were logarithmically transformed prior to analysis. Spearman rank correlation was performed to test associations between Δ_viable_ and physical-chemical parameters of the effluent samples. All statistical tests were performed with XLSTAT 2010.4.

## Abbreviations

EMA: Ethidium monoazide
PMA: Propidium monoazide
qPCR: Quantitative polymerase chain reaction
RT-PCR: Reverse transcription polymerase chain reaction
CIP: Collection de l'Institut Pasteur
VBNC: Viable but nonculturable
